# Quality of Life in Sarcoidosis

**DOI:** 10.3390/diagnostics16132079

**Published:** 2026-07-02

**Authors:** Evelyn Lynn, James Tadjkarimi, Valencia Lim, Vasileios Kouranos

**Affiliations:** 1 Interstitial Lung Disease Unit, Royal Brompton Hospital, Guy’s and St Thomas’s Foundation Trust, Sydney Street, London SW3 6NP, UK; 2 Division of Respiratory and Critical Care Medicine, University Medicine Cluster, National University Hospital, Singapore 119077, Singapore; 3 National Heart & Lung Institute, Imperial College London, London SW7 2AZ, UK

**Keywords:** sarcoidosis, quality of life (QoL), health-related quality of life (HRQoL), patient-reported outcome measures (PROMS)

## Abstract

Sarcoidosis is a multisystem disease characterised by a heterogeneous clinical presentation and variable disease course. Despite low mortality, the burden of sarcoidosis extends beyond organ involvement, with many patients reporting significant impairment in quality of life (QoL). Fatigue, chronic cough, and small fibre neuropathy (SFN) are among the most prevalent and debilitating symptoms reported by patients, often demonstrating poor correlation with conventional markers of disease activity and frequently persisting despite apparent disease control. This review examines the impact of sarcoidosis on QoL and highlights the limitations of traditional assessment tools, including imaging and pulmonary function testing, in capturing the full extent of disease burden. The role of patient-reported outcome measures (PROMs) is discussed, including generic instruments and sarcoidosis-specific tools such as the King’s Sarcoidosis Questionnaire (KSQ), Sarcoidosis Health Questionnaire (SHQ), and Sarcoidosis Assessment Tool (SAT), alongside symptom-specific measures targeting fatigue, neuropathic symptoms, and cough. Current and emerging management strategies aimed at improving QoL are reviewed, including immunosuppressive therapies, biologic agents, and non-pharmacological interventions. Despite increasing recognition of QoL as a key outcome, its integration into clinical trials remains inconsistent. Incorporating PROMs into routine clinical practice and research is essential to enable comprehensive assessment and support patient-centred care. Greater emphasis on QoL outcomes may improve therapeutic decision-making and overall disease management in sarcoidosis.

## 1. Introduction

Sarcoidosis is a multisystem granulomatous disease of unknown origin, typically presenting in individuals in early and middle adulthood [1]. The disease commonly affects patients in the 2nd to 4th decades of life, with another incidence peak observed in women mostly in the 5th decade [1,2,3,4,5]. Recent epidemiological data reported a rising prevalence over the past two decades [6]. In 2021, Cao and colleagues used data from the Global Burden of Disease study of 204 countries to estimate the Global Burden of Pulmonary Sarcoidosis and identified approximately 390,000 new cases annually and 43 million prevalent cases globally [7,8]. Population-based studies demonstrate substantial geographic variation in incidence [5,6,7,8,9]. While the prevalence of sarcoidosis is highest among those of Scandinavian origin [2,6] in Europe, the Black population has a higher prevalence compared to White Americans in the US, but no direct comparison between Europe and US has ever been made [10]. Furthermore, Black individuals experience more extra-pulmonary manifestations and demonstrate a worse prognosis with higher rates of hospitalisation and increased mortality [10,11]. A suspicion that this may be related with socioeconomic parameters rather than genetic has also been raised [12].

Given the complex symptom burden and variable disease course, quality of life has emerged as an important facet of sarcoidosis care. A wide range of symptoms may affect sarcoidosis patients’ quality of life (QoL). Fatigue, neuropathic symptoms related to small fibre neuropathy, and chronic cough are among the most commonly reported symptoms. These symptoms may develop irrespective of the extent of organ involvement. Conceptually, systemic symptoms are expected to be associated with inflammatory disease activity and may therefore improve with immunosuppressive treatment. Nonetheless, they may persist despite good control of the underlying disease. Targeted management strategies aimed at improving patient-centred outcomes are required for that purpose. This review examines the impact of sarcoidosis on quality of life, discusses validated patient-reported outcome measures used to assess this burden, and evaluates current and emerging management strategies aimed at improving patient-centred outcomes with a focus on appropriate monitoring options for clinicians involved.

## 2. Pathogenesis

It is proposed that complex interactions between host and genetic factors and environmental or infectious triggers leads to dysregulated immune response [13]. Studies propose a role for the Human Leukocyte Antigen (HLA) gene in the pathogenesis of sarcoidosis. HLA-DRB1 alleles were originally associated with Lofgren syndrome in Swedish populations [14]. Amongst Caucasians, HLA-DRB1*03, DRB1*11, DRB1*12, DRB1*14, and DRB1*15 have been identified as risks for the disease [15,16]. Among African Americans, the HLA-DQB1 alleles appear to play a more significant role in sarcoidosis susceptibility compared to HLA-DRB1 alleles [17]. While HLA-DRB1*03 has been associated with a benign disease course (my added reference above), outside the major histocompatibility complex (MHC) region, genes have also been found, suggesting that sarcoidosis phenotypes may have different genetic susceptibility and genomic distributions [18].

In sarcoidosis, symptoms affecting quality of life such as fatigue, autonomic dysfunction and small fibre neuropathy may be the end result of these inflammatory pathways which underlie the disease.

The state of chronic inflammation, often considered to be involved in such patients, is deemed to be a result of either a normal immune response to some microbial antigen or auto-antigen or a pathogenic immune response marked by sustained activation, dysfunctional regulation, and impaired resolution [13,19]. Regardless, it is clear that multiple immune pathways are involved in the pathogenesis of disease. Antigen-presenting cells (including macrophages and dendritic cells) are involved in the phagocytosis of antigens and lead to a dysregulated immune response by CD4+ T cells [20]. The resulting secretion of pro-inflammatory cytokines including Interleukins (ILs) and Interferon-gamma (IFN-γ) leads to macrophage aggregation, differentiation into epithelial cells and the formation of multinucleated giant cells or granulomas, the histopathologic hallmark of the disease [13,20,21]. Tumour necrosis factor (TNF)-α released by alveolar macrophages as well as multiple signalling pathways (such as mTOR and JAK-STAT pathway) are also involved in the pathogenesis of sarcoidosis and B cells have been implicated in the disease, as evidenced by the hypergammaglobulinemia often observed in sarcoidosis [22,23,24,25]. TNF-alpha has been found to induce neurological, haematological, metabolic and endocrine changes [26]. Studies of inflammatory vascular disease have shown increases in TNF-α, oxidative stress and inflammation-induced changes can alter neurotransmitter metabolism and can contribute to cognitive impairment [27,28]. This has led to the suggestion that TNF therapy can also result in reduced reports of fatigue by patients with sarcoidosis, though studies have shown mixed results. Trials on the impact of anti-TNF-α therapies on SAF have had mixed results [29,30,31]. These pro-inflammatory cytokines have been implicated in symptoms such as fatigue and the so-called ‘Sickness behaviour’, which is characterised by lethargy, impaired concentration, reduced motivation, and mood disturbance. Sickness behaviour appears to be the end result of the pro-inflammatory pathway where cytokines (ILs and TNF-alpha) act on the brain, leading to a syndrome characterised by lethargy, impaired concentration, reduced motivation, reduction in physical activity and dysregulation of mood [32,33]. The brain is responsible for regulating the perception of fatigue [33].

In a study to assess pro-inflammatory cytokines in post-sarcoidosis patients with fatigue, 72 patients were categorised as either being fatigued or not fatigued based on a Checklist Individual Strength subscale fatigue (CIS-f) score [34]. There was no difference in serum levels of IL-1α and IL-1β between those who reported fatigue compared to those who did not have this symptom. Another study used the Multidimensional Fatigue Inventory (MFI-20) to measure fatigue and checked levels of IL-1β in patients with sarcoidosis and in controls before and after 11–15 min of cardiopulmonary exercise testing and found no differences in IL-1β concentrations [35]. However, the authors reported pre-exercise circulating IL-1β concentrations in patients significantly correlated with fatigue severity in those patients who used immunomodulatory drugs [35], suggesting the role of treatment in how patients experience fatigue. Both studies had small numbers (study one had 72 patients, with 22 in the second study).

## 3. Sarcoidosis QoL Phenotypes

Similarly to the complexity of its pathogenesis, sarcoidosis is a heterogeneous disease with a wide range of clinical manifestations. Patients may be asymptomatic or present with a multitude of symptoms depending on the organs involved and possibly the degree of underlying inflammatory burden. The clinical disease course and outcome may also vary considerably. In many individuals the disease follows a self-limited course lasting two to three years, whereas approximately 30% of patients develop chronic disease requiring prolonged treatment [2]. Overall mortality associated with sarcoidosis is relatively low, with approximately 5% of patients dying from disease-related complications [36,37]. Mortality is most frequently associated with advanced pulmonary disease or cardiac involvement, while pulmonary hypertension is an independent predictor of poorer outcomes [38,39].

**Figure 1:**
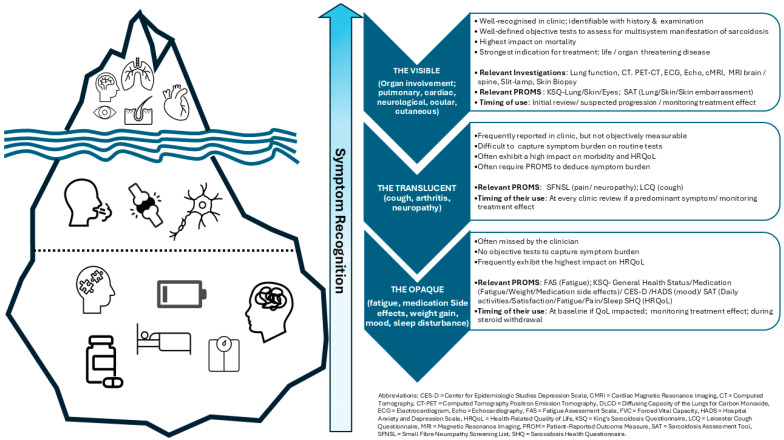
The iceberg model of sarcoidosis.

Clinicians may sometimes face potentially life-threatening organ involvement in sarcoidosis, but the majority of patients with sarcoidosis experience a constellation of systemic disease symptoms, including fatigue (40%), myalgia and arthralgia (20%), cough and eye symptoms (20%), that substantially affect their quality of life [12,40,41,42]. Such symptoms may be associated with disease activity with or without major organ involvement, but many patients continue to report symptoms even after disease remission [40]. Consequently, the burden of sarcoidosis extends beyond organ involvement alone. The most prominent and easily identifiable symptoms of multisystem sarcoidosis organ involvement profit from a wide range of available tests easily performed to confirm that. However, more challenging manifestations such as fatigue and mood/cognitive disturbance can often remain unexplored or misunderstood, in part due to the need for better tools to define and measure these entities. In this respect, sarcoidosis mirrors the classic ‘iceberg’ model of illness, where clinical attention focuses on the visible fraction while the larger submerged symptom burden which dominates the patients’ day-to-day experience can remain unexplored (Figure 1).

### 3.1. Fatigue

Fatigue is one of the most common and debilitating symptoms reported by patients with sarcoidosis. Studies suggest that between 70% and 90% of patients experience fatigue that significantly affects their quality of life [26,43,44]. Sarcoidosis-associated fatigue (SAF) is often independent of the extent and severity of organ involvement [45,46]. It is vital that clinicians routinely assess fatigue during consultations. As described earlier, the current understanding of the pathophysiology of SAF is that it is multifactorial and may involve both inflammatory and non-inflammatory pathways. A recent study in Poland found elevated levels of Interleukin-6 (IL-6) in patients with fatigue despite being in clinical remission from sarcoidosis, suggesting low-level inflammation can account for SAF [47].

When fatigue is reported, contributing causes and/or comorbidities should be explored (Table 1). Anaemia, iron deficiency, thyroid dysfunction, and sleep disordered breathing are known contributors to fatigue [26]. Low mood, depression and anxiety also need to be explored, as these are relatively common among patients with SAF [43,48]. Other inflammatory conditions including concomitant connective tissue disease and side effects from medications should also be considered when evaluating SAF. Fatigue is traditionally assessed and qualified using the Fatigue Assessment Scale (FAS), a questionnaire that covers both mental and physical fatigue [47].

### 3.2. Small Fibre Neuropathy

Small fibre neuropathy (SFN) is a cytokine-mediated, non-granulomatous complication increasingly recognised in sarcoidosis [49,50]. It is associated with neuropathic symptoms such as numbness, tingling burning sensation and sensitivity to touch as well as features of dysautonomia [49,50]. SFN results from damage to thinly myelinated Aδ fibres and unmyelinated C fibres, leading to sensory and autonomic dysfunction [50]. SFN may be idiopathic or occur in association with underlying disease activity. Patients with SFN will often be referred to neurologists for exclusion of peripheral nervous system involvement. It remains uncertain whether SFN can be caused by granulomatous inflammation and therefore be a target of immunosuppressive treatment. Often pain specialists may get involved in order to help with the management of pain that often challenges these patients [50].

Up to 60% of patients with sarcoidosis report symptoms suggestive of SFN, although the condition is likely under-recognised and under-diagnosed [43,51]. SFN-associated symptoms include paresthesia, neuropathic pain, diaphoresis, orthostasis, gastrointestinal dysmotility and palpitations. Currently, no universally accepted gold standard diagnostic criteria exist for SFN. Screening tools such as the Small Fibre Neuropathy Screening List (SFNSL) provide a validated self-administered questionnaire for identifying sarcoidosis patients with possible SFN [51,52]. To confirm the diagnosis, characteristic symptoms without large-fibre involvement combined with objective tests such as skin biopsy, corneal confocal microscopy, quantitative sudomotor axon reflex test (QSART) and thermal threshold testing are required [50,53,54]. While screening tools for SNF can be performed in the setting of a respiratory clinic, patients who develop symptoms suggestive of SFN should undergo specialist neurological evaluation, as confirmation of SFN requires dedicated neurological investigations [49].

### 3.3. Chronic Cough

Chronic cough is a frequently reported symptom in patients with pulmonary sarcoidosis and can be a major contributor to impaired QoL. Between 30% and 50% of patients may experience persistent cough during the course of their disease [30]. The pathophysiology is multifactorial and may include granulomatous inflammation of the bronchial mucosa, airway distortion from parenchymal disease, bronchial hyperactivity, upper airway involvement and even more rare causes such as involvement of neural pathways. Gastroeosophageal reflux may an independent contributor to cough that should be evaluated by clinicians. Importantly, cough severity often correlates poorly with radiographic findings or pulmonary function tests, and may persist even when inflammatory disease activity appears controlled. Persistent cough can substantially impair quality of life by disrupting sleep, limiting social interaction, and contributing to psychological distress [55,56,57,58].

## 4. Measuring Quality of Life in Sarcoidosis

Assessing the impact of sarcoidosis on QoL can be challenging to quantify. The usual tools used to assess organ involvement and monitor disease activity, including imaging and pulmonary function testing, often correlate poorly with patient-reported symptoms and overall quality of life [41,59]. Traditional serological biomarkers such as Serum Angiotension Converting Enzyme (ACE) and soluble Interleukin-2 receptor (IL-2R) are measurable, but their role remains largely supportive in the diagnostic and monitoring process as titres can vary widely between individuals [60]. Similarly, their values do not reliably track with other measures of QoL [61,62].

To provide meaningful measurement of HRQoL in sarcoidosis, patient-related outcome measures (PROMs) have been increasingly adopted in research and clinical practice. PROMs were first described in the mid-20th century, exemplified by the Cornell Medical Index. These standardised questionnaires enable patients to report symptoms, functional limitations and perceptions of health, offering insight into the burden of disease and HRQoL [63]. In clinical trials, these tools allow for evaluation of treatment effects from a patient’s perspective, and enable standardised outcome comparisons across studies [30,64,65,66].

The development and validation of PROMs should be based on established methodological criteria such as Consensus-Based Standards for the Selection of Health Measurement Instruments (COSMIN) framework [67]. COSMIN focuses on key measurement properties such as validity (whether the instrument reliably measures what it was intended to measure, and the measured aspects are truly relevant to the individual completing it), reliability (whether the results are reproducible and consistent) and responsiveness (whether the instrument can accurately detect meaningful change over time) [67].

A related concept is the Minimal Clinically Important Difference (MCID), defined as the smallest change in a PROM score that patients perceive as beneficial or meaningful, reflecting the ‘responsiveness’ measure by the COSMIN framework. Establishing the MCID not only distinguishes statistically significant results from those that matter to patients’ experience but also provides a benchmark for interpreting whether an intervention has a tangible impact on QoL. Without a validated MCID, clinical interpretation of changes in PROM scores remains ambiguous, making it difficult to discern whether observed differences represent true improvements from the patient’s perspective and/or guide treatment decisions [52,68,69]. Recognising the importance of PROMs, the World Association of Sarcoidosis and Other Granulomatous Disorders (WASOG) recommended in 2024 that all sarcoidosis research studies include HRQoL measurements [70]. Despite that, effective use of PROMs in sarcoidosis remains complex due to the broad impact of disease, multi-organ involvement, and heterogeneous symptoms [40].

### 4.1. Patient-Related Outcome Measures (PROMs)

#### 4.1.1. General PROMs

Selecting appropriate PROMs in sarcoidosis can be challenging due to the multisystem nature of the disease and constellation of symptoms [40]. PROMs originally developed for use in other respiratory diseases have been adapted and validated for use in sarcoidosis. These include the St George’s Respiratory Questionnaire (SGRQ) [71], the EuroQol 5-Dimension 5-Level (EQ-5D-5L) [72] and 36-Item Short Form Survey (36-SF) [73]. Although these tools are useful for assessing general health and have been validated for use in sarcoidosis, they may fail to capture the diverse symptom burden experienced by this group of patients and issues specifically related to sarcoidosis, limiting interpretability and thus failing to recognise response to treatment [74,75].

#### 4.1.2. Sarcoidosis-Related PROMs

To address this issue, several sarcoidosis-specific PROMs have been developed and validated for use in clinical practice and to provide surrogate measures of efficacy in clinical trials. These include the Sarcoidosis Health Questionnaire (SHQ) [76], the King’s Sarcoidosis Questionnaire (KSQ) [77], and the Sarcoidosis Assessment Tool (SAT) [68].

The SHQ features 29 items across three domains (Daily Living, Physical, and Emotional Functioning) developed to capture sarcoidosis patients’ satisfaction with life [76]. This tool demonstrates good internal consistency and can distinguish HRQoL differences based on the extent of organ involvement [76]. It was incorporated into the SARCORT study as a secondary endpoint and has also been successfully validated in a number of different languages and ethnic cohorts, including European, Turkish and Japanese [59,65,74,78]. Nonetheless, the instrument fails to address symptoms such as fatigue or cutaneous involvement, which commonly affect those with sarcoidosis [75].

SHQ [76] evaluates daily functioning, physical functioning, and emotional functioning and was developed in consultation between patients and experts [76]. It has been validated across cultures [78,79]. However, it is insensitive to changes in specific areas such as fatigue or skin changes [75].

The KSQ is a more recent (2013) 29-item questionnaire considered a current standard for pulmonary sarcoidosis [77]. Five modules address both general health status and specific organ involvement (lung, skin, eye) as well as the impact of treatment. The KSQ showed high internal consistency (Cronbach’s α ~0.8–0.9 across domains) and strong construct validity in its development cohort [77]. Baughman et al. have also demonstrated its response to clinical change with MCIDs proposed (8 points in General Health, 4 in Lung) [69] to aid in the assessment of clinical trial outcomes. A 2025 Delphi study related to pulmonary sarcoidosis clinical trial endpoints achieved consensus agreement reaching 90% that PROMs were clinically important endpoints to any future trials, and ranked KSQ domains for Lung and General HRQoL highest of any PROM [80]. Many recent trials have now integrated the KSQ into their secondary outcomes, including the PREDMETH and EFZO-FIT studies [64,66,81].

The newest tool, the SAT, is a more comprehensive, sarcoidosis-specific instrument with 51 items across 8 domains. The modular structure assesses physical functioning, satisfaction with daily activities, fatigue, pain, sleep disturbance, as well as organ-specific modules related to lung and skin involvement [68]. Notably, it was validated in a treatment trial setting, enabling determination of MCIDs for each domain, which supports its responsiveness to changes and usefulness as an endpoint in clinical trials. However, completion can be quite time-consuming, making it somewhat impractical in the clinical setting (5–10 min) [75]. The questionnaire is also not applicable to all organ-specific manifestations, and it has not been translated or cross-validated in other languages [75]. This tool has been found to have good to excellent internal consistency (Cronbach’s α ≥ 0.87 for each domain) and construct validity, though test–retest reliability and data on magnitude of change after treatment are unclear [68].

#### 4.1.3. Symptom-Specific PROMs

In addition to disease-specific HRQoL instruments, symptom-specific PROMs are often used to evaluate common sarcoidosis symptoms such as fatigue, neuropathic pain and cough.

The Fatigue Assessment Scale (FAS) is a one-dimensional, 10-item questionnaire that evaluates both physical and psychological aspects of fatigue [82,83]. Initially used to measure fatigue amongst the general population, the FAS was later validated for use in sarcoidosis and is now the most widely used fatigue PROM in sarcoidosis research, having featured in most treatment trials of the last decade [44]. Patients were not involved in the development of the tool [75]. It is brief, easy to complete and validated for use in this population [44]. The questionnaire measures both physical and mental fatigue but it remains uncertain if the scale captures all aspects of fatigue in sarcoidosis specifically [75]. It has been found to have good internal consistency and construct validity with a good test–retest reliability over 1 week [82]. An MCID of 4 points improvement in the FAS has been established, allowing for evaluation of treatment-related improvement in fatigue [82].

The Small Fibre Neuropathy Screening List (SFNSL) is a 21-point instrument specifically developed to identify SFN symptoms in sarcoidosis [51]. It captures sensory symptoms including chronic pain, dysesthesia and thermal sensitivity loss that significantly impact individuals’ QoL. It does not, however, assess autonomic symptoms and has not been validated across cultures. The SFNSL demonstrates excellent internal consistency (α ~0.90) and good validity in sarcoidosis populations with an MCID of approximately 3.5 points over a clinically relevant change over a 6-month period, making it a reliable screening tool for neuropathic involvement. SFNSL is primarily used to identify patients who may need further diagnostic evaluation, as it underscores the burden of SFN. There are no studies looking at test–retest validity, change following treatment or change over time [75].

Chronic cough is another symptom that may substantially affect QoL in sarcoidosis [40]. The Leicester Cough Questionnaire (LCQ) is a validated 19-item cough-specific survey assessing three domains of chronic cough (Physical, Psychological, Social) [84]. This instrument has been translated and validated in sarcoidosis cohorts in a number of languages, showing excellent reliability and correlation with general health status measures [74]. An MCID of approximately ≥1.3 points has been proposed based on studies in populations with chronic cough, but no specific threshold for sarcoidosis has been validated [49].

While PROMs remain a useful tool in the field of sarcoidosis research and in the clinical care of patients, there is lack of data on responsiveness, effect size, and magnitude of change after treatment even for the most widely used questionnaires. Table 2 summarises the strengths and weaknesses of PROMs with guidance on the timing of the performance and their suitability. It is important that more work is done to address existing gaps and further validate existing PROMs in sarcoidosis. With use of online PROMs there is the potential to facilitate early detection deterioration and facilitate treatment adjustments.

## 5. Managing Quality of Life Impairments in Sarcoidosis

In 2021, the ERS taskforce committee for clinical practice guidelines for treatment in sarcoidosis included QoL impairment as a key therapeutic goal following the original development of the Wells’ Law in sarcoidosis [49,85]. Well’s Law, first proposed at the World Association of Sarcoidosis and Other Granulomatous (WASOG) meeting, suggests that treatment should be initiated in patients with sarcoidosis where there is fear of danger and impaired quality of life [85]. Patient surveys further highlight that symptoms, including SAF and SFN, are often a priority for patients seeking treatment for quality of life purposes [40]. Treatments would be divided into those targeting systemic inflammation and specific inflammatory pathways, and those that are more symptom-specific.

In individuals with active sarcoidosis, glucocorticoids remain first-line therapy and are associated with improved FAS scores. The SARCORT trial compared high-dose 40 mg versus low-dose 20 mg prednisolone in a total of 86 patients and reported improvements in FAS scores in both groups at 6 and 18 months [65]. However long-term glucocorticoid use is associated with significant adverse effects such as weight gain, diabetes, hypertension, obesity, osteoporosis and glaucoma. The recent PREDMETH trial, a 24-week randomised non-inferiority study comparing methotrexate with prednisolone, showed similar results with improvement in FAS scores in both treatment arms, suggesting that methotrexate can be an effective alternative while avoiding adverse effects of glucocorticoids [66]. Infliximab, a tumour necrosis factor-alpha (TNF-α) inhibitor used to treat refractory sarcoidosis, has also been reported in small case series to improve fatigue [26]. Evidence for its use for QoL issues remains limited. Notably, Baughman and colleagues found that, despite improved forced vital capacity compared with placebo, no statistically significant differences in SGRQ scores were evident between groups in a multicentre phase two trial [30]. Non-pharmacological interventions may also provide benefits. The TIRED trial demonstrated that a 12-week online mindfulness-based cognitive behavioural therapy programme resulted in a mean reduction of 4.5 in the FAS scores of the treatment group [86].

Although clinical trials specifically targeting fatigue in sarcoidosis are limited, several pharmacological interventions have shown promising results, especially with neurostimulants. Dexmethylphenidate hydrochloride was evaluated in a double-blind, randomised, placebo-controlled, crossover trial involving only 10 patients, which showed a six-point reduction in FAS scores compared with placebo [87]. Similarly, armodafinil studied in a similarly designed controlled trial with 15 patients demonstrated a median improvement of 4 points in the FAS score after 8 weeks of treatment [87,88]. The sample sizes in both studies are small and larger-scale randomised control trials are needed to confirm reproducible results. A summary of current studies that have shown an impact of FAS scores can be found in Table 3.

Management of SFN remains challenging. Symptoms are usually refractory to conventional immunosuppressive treatment options such as glucocorticoids and/or methotrexate [89]. Symptomatic treatment typically involves medications for neuropathic pain such as amitriptyline, duloxetine, pregabalin and gabapentin [90]. A case series from the Cleveland clinic showed subjective improvement in neuropathic symptoms in 74% of patients treated with intravenous immunoglobulin, infliximab or both [48,50]. Although promising, these retrospective findings need to be interpreted with caution due to a lack of control group. In a Dutch cohort study, results showed that although infliximab reduced inflammatory activity on PET scan, it did not improve SFNSL scores [91]. This highlights the disconnect between objective imaging or biomarker response and PROM outcomes as addressed by the authors as well [91]. Emerging therapies are also being investigated. ARA 290, an erythropoietin analogue, demonstrated a greater mean reduction in the SFNSL score in the treatment group versus placebo group (12.2 versus 3.8, *p* = 0.005) in patients with sarcoidosis and associated SFN [92]. Small case series have reported improvement in SFN symptoms with Tocilizumab, an anti-IL6 monoclonal antibody, in patients refractory to other immunosuppressive therapies [93]. While these are promising results, larger multicentre studies with longer-term follow-up are needed to make definitive clinical recommendations.

Management of sarcoidosis-related chronic cough remains challenging in the absence of dedicated randomised controlled trials. The initial step should be to identify and address any reversible underlying contributor, including active granulomatous airway inflammation, gastro-oesophageal reflux disease, postnasal drip, or concomitant asthma. Where cough persists despite adequate systemic immunosuppressive therapy, inhaled corticosteroids (ICSs), such as budesonide, represent a pragmatic empirical option that may reduce localised bronchial mucosal inflammation without the systemic adverse effects of prolonged oral corticosteroid use, though evidence in this setting is largely anecdotal and extrapolated from other airway diseases. For patients with cough refractory to steroids, second-line immunosuppressive therapies and even TNF-α inhibition with infliximab may confer broader anti-inflammatory benefit, consistent with its established role in refractory pulmonary and extrapulmonary sarcoidosis; isolated cases of improvement in cough have been reported in this context. Cough-suppressant agents, including low-dose opioids (e.g., codeine) and the P2X3 receptor antagonist gefapixant, may also be considered in line with general chronic cough guidelines, though these have not been formally evaluated in sarcoidosis. The Leicester Cough Questionnaire (LCQ) should be incorporated into clinical follow-up as a standardised tool to monitor cough burden and treatment response. The absence of robust trial data in this area represents an important unmet need in sarcoidosis care.

Beyond symptom-targeted treatment, immunosuppressive therapy has also been shown to improve general QoL scores. In the PREDMETH study, treatment with both prednisolone and methotrexate was significantly associated with improvements in the KSQ lung score and the EQ-5D health index [66]. Preliminary results from the phase 3 trial (EFZO-FIT) evaluating Efzofitimod, a novel immunomodulator targeting neuropilin 2, showed significant improvement in the King’s Sarcoidosis Questionnaire (KSQ)-Lung score despite failing to meet its primary endpoint of oral corticosteroid dose reduction [64].

Although numerous emerging targeted therapies, including regimens such as tofacitinib, are being investigated for multisystem sarcoidosis, QoL outcomes are not consistently included as primary endpoints [94]. As a result, the potential impact of these therapies on patient-centred outcomes remains uncertain.

Monitoring QoL longitudinally is an essential component of sarcoidosis management [57]. Traditionally, disease extent and physiological burden are assessed at clinic review using conventional tools such as the WASOG instrument tool, pulmonary function tests, serum biomarkers (such as ACE, IL-2R) and radiographic metrics. While these modalities provide a basic understanding of the disease involvement and activity, growing evidence suggests that they frequently underestimate the true impact of the disease, especially the psychosocial impact that is of paramount importance to patients. There is often a significant disconnect between stable physiological parameters such as lung function, laboratory tests and debilitating symptoms like chronic fatigue and pain experienced by patients [63].

Routine integration of patient-reported outcome measures (PROMs) into standard clinical workflows is increasingly advocated to bridge this assessment gap, detect subclinical complications and guide personalised treatment strategies. Several expert groups now recommend incorporating validated PROMs like the FAS or KSQ into routine clinical practice, offering a more holistic, multidimensional evaluation of disease burden and supporting a patient-centred approach to sarcoidosis management [63].

There are, however, several operation barriers to the widespread use of PROMs in real-work clinical practice. The main reasons include the lack of consensus on frequency of administration, high-volume clinics with little capacity to implement, and the need for prospective validation of Minimal Clinically Important Differences in the different PROMs [95]. In this current age where artificial intelligence is harnessed to advance medical practice, perhaps integrating PROMs into electronic health records and developing machine learning frameworks would be the way forward for more personalised, symptoms-directed care for sarcoidosis [96].

Looking ahead, there is a compelling case for embedding patient-reported outcomes as co-primary or primary endpoints in sarcoidosis trials, particularly as the therapeutic landscape expands to include targeted agents such as JAK inhibitors and anti-IL-6 therapies. Current trials have largely treated QoL measures as secondary endpoints, limiting the ability to make patient-centred treatment decisions based on trial data. Future instrument development should also prioritise the assessment of cognitive impairment and sleep disturbance, which remain inadequately captured by existing PROMs. As the field increasingly embraces data science approaches, machine learning models trained on longitudinal PROM data may offer the potential to predict deterioration and guide treatment escalation in individual patients. Achieving these goals will require international collaboration, harmonisation of PROM selection across trial networks, and sustained advocacy for patient-centred endpoints at regulatory and guideline levels.

## 6. Summary

Assessment of patients with sarcoidosis includes physiological measures (lung function tests), serum markers of disease activity (ACE, IL-2R) and imaging to evaluate the extent of organ involvement and disease activity. However, these measurements in isolation do not fully capture disease burden experienced by patients. Symptoms such as fatigue, chronic cough and small fibre neuropathy frequently persist despite control of inflammatory disease activity and may profoundly affect daily functioning and well-being. Incorporating patient-reported outcome measures into routine clinical practice and clinical trials provides an opportunity to better understand and address these challenges.

## Figures and Tables

**Table 1:** Differential causes of fatigue in sarcoidosis.

Causes	Relevant Examples	Clinical Considerations
**Granulomatous inflammation**	Active pulmonary or extra-pulmonary disease	Consider other markers of disease activity: - worsening respiratory symptoms - systemic features such as fever or weight loss - other organ involvement Review: - serum inflammatory markers - serial lung function tests - Imaging (interval HRCT may help if advanced disease) - 18F-FDG-PET remains the gold standard for disease activity
**Sleep disorders**	Obstructive sleep apnoea Restless legs syndrome	Screen with Epworth scale (ESS) Consider overnight oximetry or sleep polysomnography (if ESS ≥ 10). Assess for restless leg syndrome: - serum ferritin, iron studies - renal function, electrolytes - fasting glucose, HbA1C - Vitamin B12/folate
**Psychological and cognitive factors**	Depression Anxiety	Screen with PROMs (CES-D, HADS) Refer to psychology/psychiatry accordingly
**General medical co-morbidities**	Anaemia	Check: - Iron studies - B12/folate - Exclude chronic anaemia Consider referral to haematology to exclude Lymphoproliferative disorders
**Endocrine or metabolic disorders**	Hypothyroidism Diabetes mellitus Calcium metabolism abnormalities	Routine laboratory screening: - Thyroid function testing - Fasting glucose, HbA1C - Serum calcium, 24 urine calcium, PTH, vitamin D levels
**Medication-related fatigue**	Corticosteroids 2nd-line agents Biologic therapies Non-sarcoidosis specific medications	Review treatment history carefully including both sarcoidosis specific and non-sarcoidosis specific medications Exclude adrenal insufficiency Consider increasing folic acid dose for patients on Methotrexate Routine laboratory screening required including: - Thyroid function testing - Fasting glucose, HbA1C - Serum calcium, 24 urine calcium, PTH and vitamin D levels
**Other comorbidities**	Chronic opportunistic infection Malignancy Concomitant CTD	Consider: - Fungal markers - Inflammation markers, autoimmune screen - Bronchoscopy if infectious driver - 18F-FDG-PET if necessary

Abbreviations: HRCT = High-Resolution Computed Tomography, 18F-FDG-PET = 18F-fluoro-2-deoxy-d-glucose Positron Emission Tomography, HbA1c = Haemoglobin A1c, PROMs = patient-reported outcome measures, CESDS = Center for Epidemiological Studies Depression Scale, HADS = Hospital anxiety and depression scale, PTH = Parathyroid hormone.

**Table 2:** Which PROMs to use in different scenaria.

Clinical Scenario	Recommend PROMs	MCID	Strengths	Limitations
**Initial assessment** *Identifying prevailing symptom burden*	KSQ FAS SAT SHQ	KSQ-GH: >8, KSQ-L > 4 lung module FAS > 4 SAT 2–5 points depending on module	- Establish baseline burden - Allow for longitudinal comparison (treatment response) - KSQ available in multiple languages - Well validated with good internal consistency	- No all-encompassing PROM for rarer manifestations of sarcoidosis - No MCID for SHQ
**Change in respiratory symptoms** *Increased cough/breathlessness*	KSQ-L KSQ-GH	KSQ-L > 4	- 2025 Delphi endorsement for use as endpoint in pulmonary sarcoidosis	- Limited data detailing effect of change in treatment
**Reviewing treatment** *(every 6–12 months)*	KSQ-Medication KSQ-GH	KSQ-GH: >8	- Allows for longitudinal comparison (treatment response)	- No MCID for KSQ-Medication - Captures under-recognised treatment burden
**Unexplained Fatigue** *(at baseline and/or during monitoring)*	FAS SAT ESS	FAS: 4 points SAT: 3 points ESS score of ≥10 should trigger sleep review	- FAS: Well validated, high internal consistency	- Patients not involved in original development
**Assessing extra-pulmonary sarcoidosis** *(at baseline and during monitoring if symptoms suggestive)*	KSQ-Skin KSQ-Eye SAT-Skin Skin stigma	KSQ > 8 points SAT: 2–5 points depending on module	- Targeted assessment of skin and ocular involvement - SAT modules validated across multiple sarcoidosis cohorts	- Limited normative data for extrapulmonary modules - Less extensively validated than pulmonary counterparts
**Suspected small fibre neuropathy** *(guided by symptoms,* *at baseline and/or during monitoring)*	SFNSL	≥3.5 points (over 6-month period)	- Captures chronic pain and dysesthesia. - Excellent internal consistency	- May miss autonomic symptoms
**Psychological burden** *(guided by symptoms,* *at baseline and/or during monitoring)*	CES-D HADS	CES-D ≥ 9 points HADS ≥ 1.5–2 points	- CES-D widely validated in chronic disease populations including sarcoidosis - HADS distinguishes anxiety from depression, both prevalent in sarcoidosis - Brief and patient-friendly; low response burden	- No HADS MCID specific to sarcoidosis
**Chronic Cough**	LCQ	≥1.3 points	- Validated in multiple studies	- MCID not specific to sarcoidosis

**Abbreviations:** CES-D, Center for Epidemiologic Studies Depression Scale; ESS, Epworth Sleepiness Scale; FAS, Fatigue Assessment Scale; HADS, Hospital anxiety and depression scale; HRQoL, health-related quality of life; KSQ, King’s Sarcoidosis Questionnaire; KSQ-GH, King’s Sarcoidosis Questionnaire General Health module; KSQ-L, King’s Sarcoidosis Questionnaire Lung module; LCQ, Leicester Cough Questionnaire; MCID, Minimal Clinically Important Difference; PROM, patient-reported outcome measure; QoL, quality of life; SAT, Sarcoidosis Assessment Tool; SFNSL, Small Fibre Neuropathy Screening List; SHQ, Sarcoidosis Health Questionnaire.

**Table 3:** Summary of studies with positive impact on FAS.

Trial/Study	Treatment	Impact on FAS Score
SARCORT trial	High-dose (40 mg) vs. Low-dose (20 mg) Prednisolone	Improvements in both groups at 6 and 18 months
PREDMETH trial	Methotrexate vs. Prednisolone	Similar improvements in both treatment arms at 24 weeks
Small RCT (10 patients)	Dexmethylphenidate hydrochloride	6-point reduction compared to placebo
Small RCT (15 patients)	Armodafinil	4-point median improvement at 8 weeks
TIRED trial	Online Mindfulness-Based CBT	Mean reduction of 4.5 points in the treatment group

## Data Availability

No new data were created or analyzed in this study.

## Abbreviations

The following abbreviations are used in this manuscript:

QOL	quality of life
SFN	small fibre neuropathy
PROMs	patient-reported outcome measures
KSQ	King’s Sarcoidosis Questionnaire
SHQ	Sarcoidosis Health Questionnaire
SAT	Sarcoidosis Assessment Tool
SAF	Sarcoidosis-associated fatigue
SFNSL	Small Fibre Neuropathy Screening List
HRQoL	health-related quality of life
COSMIN	Consensus-Based Standards for the Selection of Health Measurement Instruments
MCID	Minimal Clinically Important Difference
WASOG	World Association of Sarcoidosis and Other Granulomatous Disorders
SGR	St George’s Respiratory Questionnaire
EQ-5D-5	EuroQol 5-Dimension 5-Level
TNF-α	tumour necrosis factor-alpha
36SF	36-Item Short Form Survey
FAS	Fatigue Assessment Scale
LCQ	The Leicester Cough Questionnaire
HRCT	High-Resolution Computed Tomography
18F-FDG-PET	18F-fluoro-2-deoxy-d-glucose Positron Emission Tomography
HbA1c	Haemoglobin A1c
CESDS	Centre for Epidemiological Studies Depression Scale
HADS	Hospital anxiety and depression scale
PTH	Parathyroid hormone
